# Ringworm

**Published:** 1893-08

**Authors:** 


Ringworm: Its Constitutional Nature and Cure. By J. Compton Burnett, M. D. Philadelphia: Bœricke & Tafel, 1892. Price, 50 cents.

Dr. Burnett is so well known for his charming monographs that the announcement of any new creation of his pen will cause the profession to at once give attention to what he has to say.

In the present essay he treats of ringworm in his well-known and fascinating style and comes to the following conclusions, which are here stated in his own words: "(1) That it is a constitutional complaint. (2) That it is generated by the together-being of numbers of young people in close spaces—*i. e.*, by their personal emanations, or anthropotoxine. (3) That it is, so to speak, *sub-tuberculosis.* (4) That it is curable by its pathological *simillimum*, here termed Bacillinum, in high potency, internally and infrequently administered. (5) That the mycosis is merely the concomitant external manifestation of the disease and not the disease itself. (6) That the external treatment of the disease is irrational, unscientific, and probably harmful to the patient. (7) That it is commonly bred in schools. (8) That truly healthy children cannot catch it because the fungus cannot grow upon such. (9) There is, therefore, no reason why a ringwormy child should be excluded from school life or the company of its fellows in home life. (10) Finally, that the trichophyton of ringworm is to ringworm what the bacillus of Koch is to tuberculosis—the trichophyton and the bacillus being moreover nearly related to one another."

